# Commentary: Dithiothreitol (DTT), When Used as Biofilm Detaching Method to Diagnose Implant-Associated Infections, Does Not Affect Microorganisms' Viability, According to the Current Literature

**DOI:** 10.3389/fmicb.2021.814945

**Published:** 2022-02-24

**Authors:** Lorenzo Drago, Carlo Luca Romanò

**Affiliations:** ^1^Clinical Microbiology Unit, Department of Biomedical Sciences for Health, Faculty of Medicine and Surgery, University of Milan, Milan, Italy; ^2^Orthopaedic Department, Monza Polyclinic—San Gaudenzio Hospital, Novara, Italy

**Keywords:** biofilm, implant related infections, orthopedic infections, diagnosis, DTT, sonication

## Introduction

Bacteria embedded in biofilms are difficult to be dislodged and identified by traditional microbiological techniques. To detect the true pathogens, disruption and demolition of the biofilms is then proposed by different means (Drago, 2017).

This commentary builds upon the recent paper by Oliva et al. (2021). The authors conducted an acute analysis of the various microbiological methods to diagnose implant-related infections, outlining the advantages and disadvantages of the various techniques today available.

However, we think some points that may have a great relevance for daily clinical activity need to be better clarified and discussed.

## DTT and Sonication are Bacterial Culture–Based Methods

The first point that we find questionable is the definition of the “dithiothreitol assay” as a “non-culture based method,” at variance with sonication, which is instead considered a “culture based” one.

Both methods are “culture-based” because they both aim at dislodging bacteria from a given sample—dithiothreitol by chemical means, sonication by physical action—with the resulting processed fluid from both procedures requiring further culture to identify the pathogen(s).

No substantial difference can be found between sonication and dithiothreitol regarding the need for microbiological examination and concerning the possible choice of the microbiological technique used to identify the pathogen (traditional culture, molecular, or other) (Drago et al., 2013; De Vecchi et al., 2016; Villa et al., 2017).

In fact, both antibiofilm processing methods require a subsequent bacterial cultural examination, which can be chosen among all of those currently and routinely available in laboratories as both sonication and dithiothreitol only provide bacteria dislodgment from the biofilms prior to culture.

Hence, in our opinion, the classification of sonication and dithiothreitol under different chapters, “culture based methods” and “non-culture based methods,” appears not correct and needs to be rectified or at least better explained by the authors.

An incorrect classification not only has an impact from didactic and scientific points of view, but it may also induce the readers to choose one technique over the other on the basis of a false difference between pretreatments, thus compromising the diagnostic process in the clinical setting.

## DTT at 0.1% Does Not Affect Microbial Viability

A second and even more important point that we think should be amended concerns the statement from the authors concerning “the toxic effect on bacterial cells, possibly misreporting the results of the DTT fluid culture and, thus, creating false negatives,” which is reported in the text and in Table 1 of the paper.

**Table 1:** Clinical use of DTT pretreatment to dislodge microorganisms in biological samples.

**Microbiological samples**	**Activity**	**References**
Sputa pretreatment in cystic fibrosis (CF)	Facilitate quantitative studies of *S. aureus* and *P. aeruginosa*	Hammerschlag et al., 1980
Sputasol (DTT 0.1%) for sputa treatment	Optimal incubation time and bacteria release from sputa	Mcclean et al., 2010
Comparison of mucolytic agent to breakdown the mucin matrix to decrease viscosity and release bacteria trapped into the sputum network	DTT is more effective than NAC with a >90% reduction in sputum elasticity and a greater number or organisms and colony size after culture	Nielsen et al., 2004
		Saraswathy et al., 2015
Osteo and joint tissue samples	Improve the diagnosis of prosthetic joint infections	De Vecchi et al., 2016
Prosthetic components: DTT vs. sonication	Improve diagnosis of PJIs	Drago, 2017
		Sambri et al., 2018
Aortic valves	Improve diagnosis in endocarditis	Rimoldi et al., 2016
Native and heart valves	Improve diagnosis in prosthetic cardiac infections	Fontana et al., 2017

The authors do not support this statement with any reference, and as far as we know, there is, in fact, no reference that dithiothreitol, when used at the concentration normally employed in the clinical setting for diagnostic purposes (0.1% or 1 g/L), has any impact on bacteria viability.

In fact, the only reference concerning an *in vitro* bacterial inhibition of DTT for *E. coli* is reported at very high concentrations of this compound (Gill et al., 1998), which are several times more than the concentrations used in the clinical setting. The authors state indeed that 0 (without DTT), 0.25, 2.0 g/L did not affect microbial yields and were not significantly different than 0.5 and 1.0 g/L experiments, respectively.

This is very similar to what can be found for sonication, which is known to have the ability to kill bacteria (Kamineni and Huang, 2019) and requires an accurate choice of the ultrasound parameters to avoid bacterial growth inhibition (Monsen et al., 2009), and even when properly used, it may still induce phenotype changes in *E. coli* that may render challenging the microbiological diagnosis (Sendi et al., 2010).

The hypothesis that dithiothreitol may have a toxic effect is clearly contradicted by the same literature that the authors cite, including the large clinical trials performed by Sambri et al. (2018) and by Kolenda et al. (2021) as well as other relevant studies (Table 1).

How can be DTT toxic for bacteria and, at the same time, increase the sensitivity of cultural examination compared with traditional tissue cultures and sonication?

Even one of the most recent studies, performed on collection strains and not on clinical isolates (*Staphylococcus epidermidis* ATCC 35984, *Staphylococcus aureus* ATCC 43300, *Escherichia coli* ATCC 25922 and *Pseudomonas aeruginosa* ATCC 53278), shows how planktonic bacteria viability after exposure to dithiothreitol is exactly the same as that found after exposure to sonication and even to NaCl 0.9% alone (Karbysheva et al., 2020).

Dithiothreitol has been effectively used for decades in the analysis of sputa for the diagnosis of broncho-pneumonia, and *Streptococcus pneumoniae* is well-known among microbiologists as one of the most labile bacteria (Cleland, 1964; Shah and Dye, 1966; Hirsh et al., 1969; Reep and Kaplan, 1972; Isenberg, 1994; Goglio et al., 1996).

It is worth noting that the concentration of dithiothreitol to diagnose pneumonia (0.1%) is the same as that used to pretreat orthopedic samples: If this concentration was toxic, it would be even more for the *S. pneumoniae*, considered one of the most difficult bacteria to grow and to keep alive due to its lability.

DTT is indeed also used to improve diagnosis of the microbiome respiratory tract as well as for viruses (Terranova et al., 2018; Yu et al., 2018).

## Discussion

Sonication and DTT are both pretreatment techniques aimed at dislodging microorganisms from biofilms to enhance the accuracy of implant- and biofilm-related infection diagnoses. Both procedures should be classified as “culture-based” as they both require cultural examination to provide their diagnostic output, that is, pathogen identification and antibiotic sensitivity analysis.

The statement “the toxic effect on bacterial cells, possibly misreporting the results of the DTT fluid culture and, thus, creating false negatives,” to the best of our knowledge, is not supported by scientific evidence: There is no demonstration of any bacterial toxicity of dithiothreitol when used at the same concentrations adopted to diagnose implant-related infections in orthopedics and cardiovascular surgery (0.1%).

The abovementioned statement may be misleading for clinicians and may have a detrimental impact on the diagnostic algorithms implemented in many laboratories and should, hence, eventually point out that this may be the case if wrong concentrations are used, exactly as happens when sonication is improperly administered.

A last remark is worth making concerning the final statement of the authors that “additional studies evaluating the role of DTT in IAIs other than PJIs are warranted.” Although the literature on applications of DTT to diagnose implant-related infections would certainly benefit from additional studies, some papers, showing the efficacy of DTT pretreatment in cardiovascular surgery (Rimoldi et al., 2016; Fontana et al., 2017), should also be mentioned.

In conclusion, there is no scientific evidence that DTT, when properly used, affects microbial viability. Biofilm- and implant-related infections are constantly looking for definitive and resolutive diagnostic approaches, and it is hence of utmost importance to pay attention when dealing with these topics, certainly controversial and to be further improved.

## Author Contributions

LD and CLR have conceived the opinion paper and collected the relevant literature data. All authors read and approved the final manuscript.

## Conflict of Interest

LD and CR are the co-inventors of MicroDTTect (a closed system containing DTT used for collection and processing removed devices and tissues) and perceive royalties.

## Publisher's Note

All claims expressed in this article are solely those of the authors and do not necessarily represent those of their affiliated organizations, or those of the publisher, the editors and the reviewers. Any product that may be evaluated in this article, or claim that may be made by its manufacturer, is not guaranteed or endorsed by the publisher.

## References

[B1] Cleland W. W.. (1964). Dithiothreitol, a new protective reagent for SH groups. Biochemistry. 3, 480–482. 10.1021/bi00892a002 14192894

[B2] De Vecchi E. Bortolin M. Signori V. Romanò C. L. Drago L. (2016). Treatment with dithiothreitol improves bacterial recovery from tissue samples in osteoarticular and joint infections. J. Arthroplasty. 31, 2867–2870. 10.1016/j.arth.2016.05.008 27282488

[B3] Drago L.. (2017). A modern approach to biofilm-related orthopaedic implant infections: advances in microbiology, infectious diseases and public health, Vol. 5. Cham: Springer International Publishing.

[B4] Drago L. Signori V. Vecchi D. Vassena E. Palazzi C. Cappelletti E.. (2013). Use of dithiothreitol to improve the diagnosis of prosthetic joint infections. J. Orthop. Res. 31, 1694–1699. 10.1002/jor.22423 23817975

[B5] Fontana C. Favaro M. Sordillo P. Sarrecchia C. Minelli S. (2017). Microbiological approach in diagnosing native and heart valves prosthesis infections. Clin. Invest. 7, 59–64. 10.4172/Clinical-Investigation.1000112

[B6] Gill R. T. Joon Cha H. Jain A. Rao G. Bentley W. E. (1998). Generating controlled reducing environments in aerobic recombinant *Escherichia coli* fermentations: effects on cell growth, oxygen uptake, heat shock protein expression, and in vivo CAT activity. Biotechnol. Bioeng. 59, 248–259. 10.1002/(SICI)1097-0290(19980720)59:2andlt;248::AID-BIT12andgt;3.0.CO;2-A 10099335

[B7] Goglio A. Callegaro A. Farina C. Fortina G. Manso E. Piacentini I.. (1996). GSDIR. L'esame colturale nella diagnostica microbiologica delle infezioni delle basse vie respiratorie. Microbiol. Med. Boll 17, 5–10.

[B8] Hammerschlag M. R. Harding L. Macone A. Smith A. L. Goldmann D. A. (1980). Bacteriology of sputum in cystic fibrosis: evaluation of dithiothreitol as a mucolytic agent. J. Clin. Microbiol. 11, 552–557. 10.1128/jcm.11.6.552-557.1980 6776135 PMC273459

[B9] Hirsh S. R. Zastrow J. E. Kory R. C. (1969). Sputum liquefying agents: a comparative in vitro evaluation. J. Lab. Clin. Med. 74, 346–352. 5799518

[B10] Isenberg H. D.. (1994). Clinical Microbiology Procedures Handbook. Washington, DC: American Society for Microbiology.

[B11] Kamineni S. Huang C. (2019). The antibacterial effect of sonication and its potential medical application. SICOT J. 5, 19. 10.1051/sicotj/2019017 PMC6572995 31204648

[B12] Karbysheva S. Luca D. Butini M. Winkler M. E. Schütz T. Trampuz M. A. (2020). Comparison of sonication with chemical biofilm dislodgement methods using chelating and reducing agents: Implications for the microbiological diagnosis of implant associated infection. PLoS ONE. 15, e0231389. 10.1371/journal.pone.0231389 PMC7141651 32267888

[B13] Kolenda C. Josse J. Batailler C. Faure A. Monteix A. Lustig S.. (2021). Experience with the use of the MicroDTTect device for the diagnosis of low-grade chronic prosthetic joint infections in a routine setting. Front Med. 8, 565555. 10.3389/fmed.2021.565555 33796542 PMC8007775

[B14] Mcclean M. Stanley T. Goldsmith C. E. Millar B. C. McClurg B. Elborn J. S.. (2010). Determination of optimum incubation time for release of bacteria from sputum of patients with cystic fibrosis using dithiothreitol (sputasol). Br. J. Biomed. Sci. 67, 89–91. 10.1080/09674845.2010.11978195 20669768

[B15] Monsen T. Lövgren E. Widerström M. Wallinder L. (2009). In vitro effect of ultrasound on bacteria and suggested protocol for sonication and diagnosis of prosthetic infections. J. Clin. Microbiol. 47, 2496–2501. 10.1128/JCM.02316-08 19535525 PMC2725697

[B16] Nielsen H. Hvidt S. Sheils C. A. Janmey P. A. (2004). Elastic contributions dominate the viscoelastic properties of sputum from cystic fibrosis patients. Biophys. Chem. 112, 193–200. 10.1016/j.bpc.2004.07.019 15572248

[B17] Oliva A. Miele M. C. Al Ismail D. Timoteo D. De Angelis F.. (2021). Challenges in the microbiological diagnosis of implant-associated infections: a summary of the current knowledge. Front. Microbiol. 12, 750460. 10.3389/fmicb.2021.750460 PMC8586543 34777301

[B18] Reep B. R. Kaplan P. H. W. (1972). The use of n-acetyl-l-cysteine and dithiothreitol to process sputa for mycological and fluorescent antibody examination. Health Lab Sci. 9, 118–124. 4553073

[B19] Rimoldi S. G. De Vecchi E. Pagani C. Zambelli A. Di Gregorio A. Bosisio E. (2016) Use of dithiothreitol to dislodge bacteria from the biofilm on an aortic valve in the operating theatre: a case of infective endocarditis caused by Staphylococcus aureus and Proteus mirabilis. Ann Thorac Surg. 102, e357–9. 10.1016/j.athoracsur.2016.03.023. 27645982

[B20] Sambri A. Cadossi M. Giannini S. Pignatti G. Marcacci M. Neri M. P. (2018). Is treatment with dithiothreitol more effective than sonication for the diagnosis of prosthetic joint infection? Clin Orthop Relat Res. 476, 137–145. 10.1007/s11999.0000000000000060 29389758 PMC5919239

[B21] Saraswathy V. V. George P. S. Jayasree K. Sujathan K. (2015). Comparative analysis of cell morphology in sputum samples homogenized with dithiothreitol, N-acetyl-l cysteine, Cytorich® red preservative and in cellblock preparations to enhance the sensitivity of sputum cytology for the diagnosis of lung cancer. Diagn. Cytopathol. 43, 551–558. 10.1002/dc.23266 25881088

[B22] Sendi P. Frei R. Maurer T. B. Trampuz A. Zimmerli W. Graber P. (2010). *Escherichia coli* variants in periprosthetic joint infection: diagnostic challenges with sessile bacteria and sonication. J Clin Microbiol. 48, 1720–1725. 10.1128/JCM.01562-09 20335421 PMC2863887

[B23] Shah R. J. Dye W. E. (1966). Use of dithiothreitol to replace n-acetyl-l-cysteine for routine sputum digestion-decontamination for the culture of mycobacteria. Am. Rev. Respir. Dis. 94, 454. 10.1164/arrd.1966.94.3.454 5331327

[B24] Terranova L. Oriano M. Teri A. Ruggiero L. Tafuro C. Marchisio P. (2018). How to process sputum samples and extract bacterial DNA for microbiota analysis. Int. J. Mol. Sci. 19, 3256. 10.3390/ijms19103256 PMC6214103 30347804

[B25] Villa F. Toscano M. De Vecchi E. Bortolin M. Drago L. (2017). Reliability of a multiplex PCR system for diagnosis of early and late prosthetic joint infections before and after broth enrichment. Int. J. Med. Microbiol. 307, 363–370. 10.1016/j.ijmm.2017.07.005 28750797

[B26] Yu F. Qiu F. Zeng T. Wang Y. Zheng Y. Chen S. Chen X. Y. (2018). Comparative evaluation of three preprocessing methods for extraction and detection of influenza A virus nucleic acids from sputum. Front. Med. 5, 56. 10.3389/fmed.2018.00056 29552562 PMC5840144

